# BSEF-Stereo: A Stereo Matching Model Based on Branching Strategy and Error Feedback

**DOI:** 10.3390/s26134318

**Published:** 2026-07-07

**Authors:** Kaicheng Li, Jinlong Yang, Chin Chi Choi

**Affiliations:** 1 School of Artificial Intelligence and Computer Science, Jiangnan University, Wuxi 214122, China; qtqs3212@126.com; 2 MTel Telecommunication Company Limited, Macao, China

**Keywords:** stereo matching, iterative optimization, branching strategy, error feedback, disparity refinement

## Abstract

Iterative stereo matching remains challenging in weakly textured regions, repetitive patterns, occlusions, and object boundaries, where ambiguous correspondence cues require broad contextual reasoning while accurate reconstruction depends on preserving local structural details. Existing recurrent updaters with a fixed receptive field struggle to balance these requirements, and their initial disparity estimates may retain local geometric inconsistencies. To address these limitations, we propose BSEF-Stereo, an iterative framework that combines adaptive recurrent updating with explicit error-feedback refinement. A channel–position attention module strengthens discriminative channel and spatial cues, while a branch-strategy gated recurrent unit uses complementary small- and large-kernel branches to preserve boundary details and aggregate context in ambiguous regions. An error-aware refinement module subsequently exploits reprojection error and image guidance to correct the initial disparity map. Experiments on Scene Flow, KITTI 2012, KITTI 2015, and Middlebury demonstrate competitive performance across synthetic, outdoor, and indoor scenes. BSEF-Stereo achieves 0.41 px EPE and 2.27% D1 on Scene Flow and a D1-all error of 1.48% on KITTI 2015. Ablation and sensitivity studies verify the complementary contributions of the three modules and support the selected design settings.

## 1. Introduction

Stereo matching is a fundamental task in computer vision, aiming to estimate dense disparity by identifying corresponding pixels between a pair of rectified stereo images. It has been widely applied in 3D reconstruction, autonomous driving, robotic perception, and scene understanding. With the rapid development of deep learning, stereo matching methods have achieved substantial progress in both accuracy and robustness. Nevertheless, accurate disparity estimation remains challenging in practical scenarios, especially in weakly textured regions, repetitive patterns, occluded areas, illumination variations, and object boundaries.

The development of deep learning has enabled convolutional neural networks (CNNs) to be widely used for end-to-end stereo matching frameworks [1,2,3], leading to notable improvements in disparity estimation accuracy. Most existing deep stereo approaches follow a cost-filtering paradigm [4,5,6,7,8]. In this framework, CNN-based feature extractors are first applied to obtain representations from stereo image pairs. These features are then used to build a 3D or 4D cost volume, which is further processed by 2D or 3D convolutional networks to aggregate matching costs and finally regress the disparity map. Despite these advantages, this paradigm still suffers from an inherent limitation. During cost volume construction and aggregation, the feature resolution is usually reduced, which may cause the loss of fine image details such as edges and texture details. Consequently, the accuracy of disparity estimation may degrade in regions containing fine structures or sharp object boundaries.

Recently, iterative optimization methods [9,10,11,12] have attracted increasing attention and achieved state-of-the-art performance on several benchmarks. These methods typically construct an all-pairs cost volume, retrieve local correlation features from this volume, and employ recurrent units to predict disparity residuals for iterative refinement. By retaining matching evidence over a broad disparity range, they avoid relying on a narrowly predefined search range. They also replace repeated cost-volume aggregation with lightweight recurrent updates, making them well suited to high-resolution disparity estimation.

However, iterative optimization methods still suffer from several limitations. First, constructing cost volumes using unimodal features or simple correlation operations may fail to fully capture complex image characteristics, such as local structures and texture variations. As a result, the extracted representations may be insufficient for reliable matching in challenging regions. Second, the iterative update mechanism is prone to converging to local optima, especially in scenes with complex textures, repetitive patterns, or low-contrast regions.

To address these limitations, we propose BSEF-Stereo, an iterative stereo matching framework built on a branching update strategy and error-feedback refinement. First, the channel–position attention module jointly models channel dependencies and spatial positional cues to enhance stereo feature representation. Second, the branch-strategy gated recurrent unit adaptively combines local details with broader contextual information during iterative disparity updating. Third, the error-aware refinement module uses reprojection error and image guidance to correct local geometric inconsistencies in the initial disparity map.

The main contributions of this work are summarized as follows. First, we introduce CPA to enhance stereo features from both channel and spatial-position dimensions, improving the discriminability of matching representations in ambiguous regions. Second, we design BGRU to combine small-kernel local detail modeling with large-kernel contextual aggregation during recurrent disparity updating. Third, we propose EAR to refine the initial disparity map by explicitly using reprojection error and image-guided structural cues. Extensive experiments and ablation studies on Scene Flow, KITTI 2012 [13], KITTI 2015 [14], and Middlebury [15] verify the effectiveness of these components and position the proposed framework within recent iterative stereo matching methods.

## 2. Related Work

### 2.1. Stereo Matching

In recent years, the rapid development of deep learning has significantly advanced stereo matching, and convolutional neural network (CNN)-based approaches have become the dominant paradigm for improving disparity estimation performance. One of the earliest attempts to introduce deep neural networks into stereo matching is DispNet [2], which formulates disparity estimation as an end-to-end learning problem. GCNet [1] constructs a 3D cost volume by concatenating left and right image features at different disparity candidates and adopts 3D convolutions for cost aggregation. PSMNet [16] incorporates spatial pyramid pooling (SPP) to capture global contextual information and employs stacked hourglass networks to refine the cost aggregation process.

Later research explored more sophisticated cost aggregation mechanisms. Based on SGM [17], GANet [18] introduces a learnable semi-global aggregation module that enables directional cost propagation and proposes a local guided aggregation layer to better preserve thin structures and object boundaries. However, these cost filtering methods are limited by the computing resolution, and it is difficult to adapt to high-resolution images. To overcome the resolution limitations of cost-filtering methods, Teed et al. proposed Recurrent All-Pairs Field Transform (RAFT) [19], which constructs a four-dimensional correlation volume for all pixel pairs and iteratively updates optical flow by retrieving correlation features with a recurrent unit. RAFT-Stereo [11] adapts this recurrent formulation to stereo matching by replacing the four-dimensional all-pairs correlation volume with a three-dimensional stereo correlation volume and using multi-level ConvGRUs for high-resolution disparity refinement. IGEV-Stereo [20] introduces geometrically encoded volumes containing geometry, context, and local match details and progressively optimizes the disparity map through iterative indexing.

More recently, research has explored several new directions to further improve stereo matching performance and generalization ability. One emerging trend is the integration of foundation models and monocular depth priors. For example, FoundationStereo [21] proposes a foundation model for stereo matching and demonstrates strong zero-shot generalization capability across different datasets. Stereo Anywhere [22] combines geometric stereo matching with monocular depth priors derived from large-scale vision models, enabling robust performance even when stereo cues are unreliable.

Another important research direction focuses on improving iterative refinement and global reasoning capabilities. MonSter [23] jointly optimizes monocular depth and stereo estimation through a cooperative refinement mechanism. Furthermore, S^2^M^2^ [24] proposes a scalable stereo matching architecture designed to handle varying resolutions and disparity ranges.

Although iterative optimization techniques have made significant progress, further improvements are still required as a single structure may not fully adapt to complex stereo matching tasks.

### 2.2. Attention-Based and Multi-Scale Feature Enhancement

Attention and multi-scale feature enhancement have been widely used to improve stereo representations in ambiguous matching regions. SENet [25] and ECA-Net [26] show that channel-wise reweighting can strengthen informative feature responses with limited computational overhead, while coordinate-style positional encoding can preserve spatial dependency cues that are important for dense prediction. In stereo matching, these mechanisms are particularly useful because weakly textured regions, repetitive patterns, occlusions, and object boundaries require both discriminative local details and broader contextual support.

Compared with methods that explicitly operate in the spectral domain, the proposed BSEF-Stereo does not perform Fourier, wavelet, or octave decomposition. Therefore, we describe the proposed design as receptive-field-aware and attention-based feature enhancement rather than as an explicit spectral method. The small-kernel and large-kernel branches in the recurrent updater are used to model complementary receptive fields: the small-kernel branch focuses on local details such as object boundaries and thin structures, whereas the large-kernel branch captures broader contextual information in weakly textured and ambiguous regions.

## 3. Method

**Figure 1:**
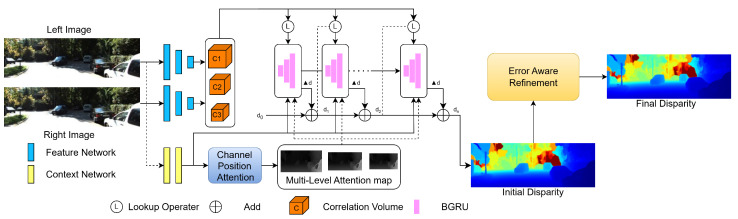
General structure of the proposed BSEF-Stereo. The model consists of two main components: an iterative optimization network and a disparity refinement network. Solid arrows indicate the main data flow, dashed arrows indicate auxiliary guidance or feedback connections. The ellipses denote repeated iterative updating steps.

This paper proposes BSEF-Stereo, an iterative stereo matching framework based on branching strategy and error feedback. As illustrated in Figure 1, the proposed model consists of two main components: an iterative optimization-based stereo matching network and a disparity refinement network. The iterative optimization network includes a multi-scale feature extraction network, a channel–position attention (CPA) module, a cost volume pyramid construction module, and a branch-strategy gated recurrent unit (BGRU) iterative updater. Specifically, the feature extraction network consists of several residual blocks with progressive downsampling and produces multi-scale feature maps at 1/4, 1/8, and 1/16 resolutions. The 1/4-resolution feature maps are used to construct the initial correlation volume, while the lower-resolution features provide contextual information for iterative disparity updating. The context network adopts a similar residual structure and outputs hidden-state features and context features for the recurrent updater. During each iteration, BGRU progressively updates the disparity estimate by integrating the cost volume, contextual features, and attention cues generated by CPA. In BGRU, the small-kernel branch focuses on local detail preservation around object boundaries and thin structures, whereas the large-kernel branch captures broader contextual information for weakly textured and ambiguous regions. The two candidate hidden states are then adaptively fused using the attention map generated by CPA. After the iterative network obtains the initial disparity map, the disparity refinement network further refines it through an error-aware hourglass aggregation module. This module takes the concatenation of the reprojection error map, the original left image, and the normalized initial disparity map as input and predicts a residual disparity map. The residual is added to the initial disparity prediction to produce the final refined disparity map.

### 3.1. Feature Extraction

**Feature network.** The feature extraction module adopts a multi-scale design consisting of several residual blocks and progressive downsampling layers. Given a rectified stereo pair $\left(I_{l},I_{r}\right)\in R^{H\times W\times 3}$, the feature extraction network produces multi-scale feature maps:

(1) $$F_{l,r}^{s}\in R^{\frac{H}{s}\times \frac{W}{s}\times C_{s}},\;s\in \left\{4,8,16\right\}$$

**Context network.** The architecture maintains consistency with the feature network, ultimately obtaining multi-level contextual features $f_{i}^{c}$ (i=1,2,3) at 1/4, 1/8, and 1/16 scales, from which the initial hidden state and contextual information can be derived:

(2) $$h_{0}^{s}=\tanh\left(f_{s}^{c}\right),$$

(3) $$c^{s}=\mathrm{ReLU}\left(f_{s}^{c}\right),$$

where $h_{0}^{s}$ denotes the initial hidden state at scale s, and $c^{s}$ denotes the corresponding context feature used by the recurrent updater.

### 3.2. Cost Volume Construction

Following common practice in iterative stereo matching frameworks [11,19,20], we construct a correlation volume using the 1/4-resolution feature maps $f_{l(1/4)}$ and $f_{r(1/4)}$ extracted from the left and right images. To capture matching information at different scales, a multi-level correlation volume pyramid is generated through successive average pooling operations.

### 3.3. Channel–Position Attention Module

**Figure 2:**
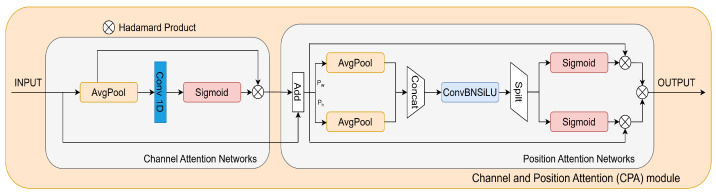
The architecture of the proposed channel–position attention (CPA) module.

Conventional cost-volume construction may treat feature channels and spatial positions uniformly, limiting the representation of informative edges and textures. Rather than defining explicit frequency bands, we enhance complementary channel and positional cues directly in the convolutional feature maps. The proposed channel–position attention (CPA) module combines channel attention with position attention to strengthen discriminative structural information for subsequent cost-volume construction and iterative disparity optimization. Its architecture is illustrated in Figure 2.

The channel-attention branch takes contextual features as input and extends the mechanism used in SENet [25]. SENet generates channel weights through two fully connected layers and a Sigmoid function, but its dimensionality-reduction operation may discard useful channel information. Moreover, explicitly modeling dependencies among all channels can be computationally inefficient. Inspired by ECA-Net [26], we therefore replace the fully connected layers with a one-dimensional convolution that captures local cross-channel interactions without dimensionality reduction. A convolution with kernel size *k* allows each channel to interact with its *k* neighboring channels, thereby controlling the interaction range used to estimate channel attention.

Given an input feature map $X\in R^{C\times H\times W}$, where *C*, *H*, and *W* denote the number of channels, height, and width, respectively, the proposed CPA module enhances stereo features from both channel- and spatial-position dimensions.

For channel attention, global average pooling (GAP) is first applied to aggregate the global spatial response of each channel:

(4) $$z_{c}=\mathrm{GAP}\left(X_{c}\right)=\frac{1}{H\times W}\sum_{i=1}^{H}\sum_{j=1}^{W}X_{c}\left(i,j\right),\;c=1,2,\ldots ,C$$

where $X_{c}\left(i,j\right)$ denotes the feature value of the *c*-th channel at spatial position (i,j), and $z_{c}$ is the corresponding channel descriptor. Thus, the global channel descriptor can be represented as $z=\left[z_{1},z_{2},\ldots ,z_{C}\right]\in R^{C}$.

Different from the conventional SENet, which uses two fully connected layers with dimensionality reduction to estimate channel weights, the proposed module removes the dimensionality reduction operation and adopts a one-dimensional convolution to model local cross-channel interactions:

(5) $$s_{c}=\sigma \left(\mathrm{Conv}1D_{k}\left(z\right)\right)$$

where $\mathrm{Conv}1D_{k}\left(\cdot \right)$ denotes a one-dimensional convolution with kernel size *k*, σ(·) is the Sigmoid activation function, and $s_{c}\in R^{C}$ denotes the channel attention weights. The kernel size *k* is adaptively determined according to the channel number *C*:

(6) $$k={\left|\frac{\log_{2}\left(C\right)+b}{\gamma }\right|}_{\mathrm{odd}}$$

where ${\left|\cdot \right|}_{\mathrm{odd}}$ denotes the nearest odd integer. In this paper, γ=2 and b=1 are used. This adaptive strategy enables the channel attention module to capture local cross-channel dependencies with a suitable interaction range.

To further preserve spatial positional information, the position attention branch encodes long-range dependencies along the height and width directions. Specifically, average pooling is performed along the horizontal and vertical directions:

(7) $$z_{h}^{c}\left(i\right)=\frac{1}{W}\sum_{j=1}^{W}X_{c}\left(i,j\right),\;i=1,2,\ldots ,H$$

(8) $$z_{w}^{c}\left(j\right)=\frac{1}{H}\sum_{i=1}^{H}X_{c}\left(i,j\right),\;j=1,2,\ldots ,W$$

where $z_{h}^{c}\left(i\right)$ and $z_{w}^{c}\left(j\right)$ encode position-aware responses along the height and width directions, respectively. These two directional descriptors are concatenated and transformed by a 1×1 convolution:

(9) $$f=\delta ({\mathrm{Conv}}_{1\times 1}\left(\left[z_{h},z_{w}\right]\right))$$

where [·,·] denotes the concatenation operation and δ(·) denotes a nonlinear activation function. The fused feature *f* is then split into two separate tensors:

(10) $$\left[f_{h},f_{w}\right]=\mathrm{Split}\left(f\right),\;\;\;f_{h}\in R^{C\times H\times 1},\;\;\;f_{w}\in R^{C\times 1\times W}$$

where Split(·) separates the fused descriptor according to the original height and width directions: $f_{h}$ corresponds to the height-wise descriptor and $f_{w}$ corresponds to the width-wise descriptor. The height-wise and width-wise attention maps are generated by:

(11) $$s_{h}=\sigma \left({\mathrm{Conv}}_{h}\left(f_{h}\right)\right)$$

(12) $$s_{w}=\sigma \left({\mathrm{Conv}}_{w}\left(f_{w}\right)\right)$$

Finally, the output of the CPA module is obtained by applying channel and position attention to the input feature:

(13) $$X_{\mathrm{out}}=X⊙s_{c}⊙s_{h}⊙s_{w}$$

where ⊙ denotes element-wise multiplication. The attention maps $s_{c}$, $s_{h}$, and $s_{w}$ are broadcast to the same spatial size as *X* during multiplication. The attention map used in the BGRU branch fusion is obtained by projecting the CPA-enhanced feature into a single-channel spatial attention map:

(14) $$A_{t}=\sigma \left({\mathrm{Conv}}_{1\times 1}\left(X_{\mathrm{out}}\right)\right)$$

### 3.4. Initial Disparity Estimation

**Figure 3:**
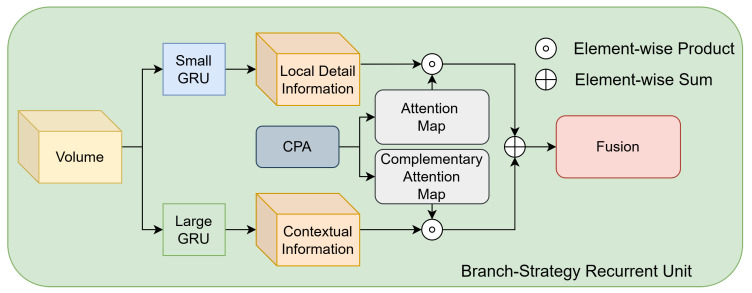
The architecture of the proposed branch-strategy gated recurrent unit (BGRU).

A recurrent updater with a single convolutional kernel cannot simultaneously capture broad contextual information and preserve fine local details. This limitation is consistent with two observations from previous studies: neural networks tend to learn low-frequency components more readily than high-frequency details [27], and repeated local aggregation can produce over-smoothing that weakens feature discriminability [28]. Consequently, boundary cues and thin structures may become less distinguishable when all regions are updated using a fixed receptive field. To address this issue, we propose a branch-strategy gated recurrent unit (BGRU). As shown in Figure 3, the iterative framework employs a three-level hierarchy operating at 1/4, 1/8, and 1/16 of the input resolution. At each iteration, BGRU integrates multi-scale correlation features, the current hidden state $h_{t}$, and attention maps generated by CPA to predict a sequence of disparity maps $\left\{d_{1},d_{2},\ldots ,d_{N}\right\}$. The initial hidden state $h_{0}$ is derived from contextual features. A two-layer disparity head then predicts the residual $\Delta d_{t}$, which is added to the current estimate $d_{t}$ to obtain the disparity for the next iteration:

(15) $$d_{t+1}=d_{t}+\Delta d_{t}$$

Finally, the disparity predictions at 1/4 resolution are upsampled to the original image resolution using a convex upsampling strategy.

**Branch-strategy gated recurrent unit.** To accommodate regions with different receptive-field requirements, BGRU employs two recurrent branches with different kernel sizes. Unlike a standard convolutional GRU [29], which uses a single kernel for hidden-state updating, the small-kernel branch focuses on local details, such as object boundaries and thin structures, whereas the large-kernel branch captures broader contextual information in weakly textured and ambiguous regions.

Given the input feature $x_{t}$ and the previous hidden state $h_{t-1}$, a standard convolutional GRU can be formulated as:

(16) $$z_{t}=\sigma \left(\mathrm{Conv}\left(\left[h_{t-1},x_{t}\right],W_{z}\right)\right)$$

(17) $$r_{t}=\sigma \left(\mathrm{Conv}\left(\left[h_{t-1},x_{t}\right],W_{r}\right)\right)$$

(18) $${\tilde{h}}_{t}=\tanh\left(\mathrm{Conv}\left(\left[r_{t}⊙h_{t-1},x_{t}\right],W_{h}\right)\right)$$

(19) $$h_{t}=\left(1-z_{t}\right)⊙h_{t-1}+z_{t}⊙{\tilde{h}}_{t}$$

In the proposed BGRU, two candidate hidden states are generated by the small-kernel and large-kernel branches, denoted as $h_{t}^{sm}$ and $h_{t}^{lg}$, respectively. The final hidden state is obtained by

(20) $$h_{t}=A_{t}⊙h_{t}^{sm}+\left(1-A_{t}\right)⊙h_{t}^{lg}$$

where $A_{t}$ denotes the attention map generated from the CPA module at the corresponding scale. As discussed above, the attention map is used to adaptively balance local detail modeling and contextual aggregation. Since the attention map assigns higher responses to object boundaries and thin structures, the small-kernel GRU branch is directly multiplied by $A_{t}$ to preserve edge and slender-object details. In contrast, the large-kernel GRU branch is multiplied by the complementary map $\left(1-A_{t}\right)$, allowing weakly textured and ambiguous regions to rely more on broader contextual aggregation.

### 3.5. Error-Aware Refinement

**Figure 4:**
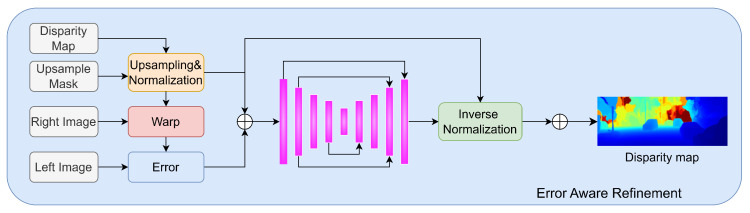
Error-aware refinement. The plus symbols denote feature fusion and residual addition at the corresponding fusion points.

The initial disparity maps generated by the iterative optimization network may still contain local geometric inconsistencies, especially around object boundaries, occluded regions, and weakly textured areas. This is because the left–right view consistency and image-guided structural information are not fully exploited during the iterative update process. To optimize the final disparity output, we implement two key improvements: (1) reusing the 1/4-resolution initial disparity predicted by the BGRU network and (2) integrating structural cues from the original left image. As shown in Figure 4, we introduce an error-aware refinement (EAR) module that exploits both reprojection error and image guidance.

Given the initial disparity map *D*, the right image $I_{r}$ is first warped to the left view using differentiable warping:

(21) $$I_{l}^{\prime }=W\left(I_{r},D\right)$$

where W(·) denotes the warping operation. The reprojection error map is then computed as:

(22) $$E=|I_{l}-I_{l}^{\prime }|$$

Compared with the signed difference, the absolute reprojection error directly reflects the photometric inconsistency between the reconstructed left image and the original left image.

The initial disparity map is normalized by the image width:

(23) $$D_{\mathrm{norm}}=\frac{D}{W_{\mathrm{img}}}$$

Then, the reprojection error map, the left image, and the normalized disparity map are concatenated and fed into an hourglass refinement network:

(24) $$\Delta D=H_{\theta }\left(\left[E,I_{l},D_{\mathrm{norm}}\right]\right)$$

where $H_{\theta }\left(\cdot \right)$ denotes the hourglass refinement network and ΔD is the predicted residual disparity. The final disparity map is obtained by:

(25) $$D_{f}=D+\epsilon \Delta D$$

Here, ϵ denotes the residual scaling coefficient and is set to 0.5 in the final model. This coefficient constrains the magnitude of the residual disparity correction, allowing EAR to refine local errors without overriding the coarse geometric structure estimated by the iterative network. The setting is supported by the residual-scaling sensitivity analysis reported in Section 4.3.

Differentiable warping reconstructs the left view from the right image and the estimated disparity, allowing photometric inconsistencies to be represented explicitly by the reprojection-error map. EAR combines this error signal with the original left image, which supplies structural guidance for correcting local disparity errors. The resulting refinement is intended to improve geometric consistency while preserving fine details and sharp boundaries.

### 3.6. Loss Function

The loss of the entire network is defined as

(26) $$L_{iter}=\sum_{i=1}^{N}\lambda^{N-i}\frac{1}{\left|\Omega \right|}\sum_{p\in \Omega }\left|d_{i}\left(p\right)-d_{gt}\left(p\right)\right|,\;\mathrm{where}\;\lambda =0.9$$

(27) $$L_{fin}=\frac{1}{\left|\Omega \right|}\sum_{p\in \Omega }\left|d_{fin}\left(p\right)-d_{gt}\left(p\right)\right|$$

(28) $$L=L_{iter}+L_{fin}$$

where $L_{\mathrm{iter}}$ and $L_{\mathrm{fin}}$ denote the iterative and final losses, respectively; $d_{i}$ is the disparity predicted at iteration *i*; $d_{\mathrm{gt}}$ is the ground-truth disparity; $d_{\mathrm{fin}}$ is the final refined disparity; λ is the exponential discount factor; and Ω is the set of pixels with valid ground truth.

The iterative loss applies temporal discounting so that predictions closer to the final iteration receive larger weights. This schedule allows early estimates to be coarse while encouraging progressively more accurate disparity updates. The final loss $L_{\mathrm{fin}}$ is an L1 loss between the refined disparity $d_{\mathrm{fin}}$ and the ground truth $d_{\mathrm{gt}}$. Their sum supervises both the intermediate disparity sequence and the final output of the refinement network.

## 4. Experiments

We evaluate BSEF-Stereo on Scene Flow [2], KITTI 2012 [13], KITTI 2015 [14], and Middlebury [15]. Together, these datasets cover diverse acquisition conditions. Scene Flow provides rendered stereo pairs with dense ground truth; KITTI 2012 and KITTI 2015 contain outdoor driving scenes captured under natural illumination and traffic conditions; and Middlebury provides high-resolution indoor scenes acquired using controlled camera setups. Because lighting changes, shadows, reflections, and low-contrast surfaces can weaken local correspondence cues, evaluation across synthetic, outdoor, and indoor data provides a broader assessment of model robustness.

### 4.1. Implementation Details

The framework is implemented in PyTorch 1.12.1 and trained on a single NVIDIA GeForce RTX 4090 GPU using AdamW with a weight decay of $1\times 10^{-5}$. All models are first pretrained on Scene Flow for 500,000 iterations using random 384×736 crops, a batch size of 2, and an initial learning rate of $2\times 10^{-4}$. For KITTI, the KITTI 2012 and KITTI 2015 training sets are combined, and the pretrained model is fine-tuned for 6000 iterations using random 320×1024 crops, a batch size of 2, and the same learning rate. We use 22 recurrent updates during training and 32 during inference. The inference iteration count, BGRU kernel sizes, and residual scaling factor ϵ are selected according to the sensitivity analyses reported after the ablation study.

### 4.2. Ablation Study

To verify the contribution of each proposed component, we conduct ablation experiments on Scene Flow and KITTI, as shown in Table 1. The baseline model obtains an EPE of 0.56 px and a D1 error of 2.61% on Scene Flow and an EPE of 0.43 px and a D1 error of 1.22% on KITTI.

**Channel–position attention (CPA) module:** We first compare the baseline with different attention variants. Channel attention reduces EPE from 0.56 px to 0.50 px on Scene Flow and from 0.43 px to 0.38 px on KITTI, showing the benefit of channel-wise feature reweighting. Position attention also improves the baseline, yielding 0.52 px EPE and 2.52% D1 on Scene Flow and 0.40 px EPE and 1.21% D1 on KITTI. These results show that both channel and positional cues contribute to stereo feature representation.

Combining the two branches in CPA further improves performance to 0.48 px EPE and 2.39% D1 on Scene Flow and 0.36 px EPE and 1.02% D1 on KITTI. The consistent gains over either branch alone indicate that channel dependencies and position-aware structural information provide complementary cues.

**Branch-strategy gated recurrent unit (BGRU):** Used independently, BGRU reduces EPE from 0.56 px to 0.47 px on Scene Flow and from 0.43 px to 0.36 px on KITTI. When applied together with CPA, replacing the conventional GRU with BGRU further reduces EPE from 0.48 px to 0.43 px on Scene Flow and from 0.36 px to 0.33 px on KITTI; the KITTI D1 error also decreases from 1.02% to 0.86%. These improvements support the use of complementary receptive fields during recurrent disparity optimization.

**Error-aware refinement (EAR):** Adding EAR independently reduces EPE from 0.56 px to 0.50 px on Scene Flow and from 0.43 px to 0.40 px on KITTI. Under the CPA-enhanced setting, replacing the conventional hourglass refinement module with EAR further reduces EPE from 0.48 px to 0.45 px on Scene Flow and from 0.36 px to 0.34 px on KITTI, while KITTI D1 decreases from 1.02% to 0.92%. The results indicate that reprojection-error feedback and image guidance provide useful cues for correcting local disparity errors.

Overall, Table 1 evaluates the proposed components under controlled combinations. CPA is examined through channel-only, position-only, and joint attention variants, while BGRU and EAR are evaluated both independently and under the same CPA-enhanced setting. The full model achieves the best results on both datasets. Relative to the baseline, it reduces Scene Flow EPE from 0.56 px to 0.41 px and D1 from 2.61% to 2.27%, corresponding to relative reductions of 26.8% and 13.0%, respectively. On KITTI, EPE decreases from 0.43 px to 0.31 px and D1 from 1.22% to 0.72%, giving relative reductions of 27.9% and 41.0%. The improvements across both average error and outlier rate indicate that the three modules provide complementary benefits rather than improving only a single metric.

### 4.3. Hyperparameter Sensitivity Analysis

We conduct sensitivity analyses for the number of inference iterations, the BGRU kernel-size configuration, and the residual scaling factor ϵ in EAR. The kernel-size and ϵ sensitivity experiments are conducted on the Scene Flow dataset. These experiments justify the selected settings from the perspectives of accuracy, outlier control, and refinement stability.

**Number of iterations:** Table 2 shows that increasing the number of recurrent updates consistently reduces errors on both Scene Flow and KITTI. The largest improvement occurs during the early iterations: increasing the count from 2 to 8 reduces Scene Flow EPE from 0.953 px to 0.495 px and KITTI EPE from 0.534 px to 0.348 px. Further iterations continue to improve the estimates, although the gains gradually diminish as the disparity converges. From 16 to 32 iterations, Scene Flow EPE decreases by another 0.041 px and KITTI EPE by 0.020 px. Because 32 iterations provide the lowest EPE and D1 values on both datasets, this setting is adopted for the final inference configuration.

**Kernel-size sensitivity:** Table 3 compares three BGRU kernel-size combinations on Scene Flow. The 1×1+1×5 configuration produces the highest EPE and >1 px error, suggesting that a strongly anisotropic large branch is less suitable for the tested scenes. Replacing the small branch with a 3×3 kernel reduces the >1 px error to 5.05%, but the EPE remains 0.42 px. The 1×1+3×3 configuration achieves the lowest EPE of 0.41 px with a comparable >1 px error of 5.08%. We therefore select this configuration because it provides the best average disparity accuracy while retaining competitive outlier performance, supporting the use of compact local and broader spatial receptive fields in the two branches.

**Residual scaling sensitivity:** Table 4 shows that the refinement scale must balance correction strength and stability. With ϵ=0.25, the model obtains 0.42 px EPE and 5.17% > 1 px error, indicating that conservative residual updates leave some errors insufficiently corrected. Increasing ϵ to 0.50 improves both metrics to 0.41 px and 5.05%. In contrast, ϵ=0.75 degrades EPE to 0.47 px and the outlier rate to 5.45%, showing that overly large corrections can disturb otherwise reliable disparity estimates. Therefore, ϵ=0.50 is adopted as the most balanced setting.

### 4.4. Computational Complexity and Efficiency Analysis

**Complexity and efficiency analysis:** Table 5 compares the parameter counts and peak GPU memory of the model variants; memory is measured on an NVIDIA GeForce RTX 3050 Ti with FP32 inference, batch size 1, 384×1248 input resolution, and 32 iterations, where HG denotes the baseline hourglass module. Replacing the baseline GRU and HG with BGRU reduces the parameter count from 13.14M to 12.95M (1.4%), while the two recurrent branches increase peak memory by only 0.017 GiB. CPA causes no measurable increase in parameter count or peak memory, confirming that its feature reweighting introduces negligible computational overhead. EAR increases the complete model to 15.29M parameters, but peak memory rises by only 0.009 GiB over the CPA–BGRU variant. Consequently, the full model requires 16.4% more parameters but only 2.1% more peak memory than the baseline. When considered together with the KITTI D1 reduction from 1.22% to 0.72% in Table 1, this result indicates that the main accuracy improvement is obtained without a proportional increase in inference memory. The runtime of 0.29 s per image pair is slower than lightweight real-time methods but remains comparable to several high-accuracy iterative approaches in the subsequent KITTI comparisons.

### 4.5. Comparisons with Other Methods

**Scene Flow dataset:** Table 6 shows that BSEF-Stereo achieves the best performance among the selected methods, with an EPE of 0.41 px and a D1 error of 2.27%. It reduces EPE by 0.68 px relative to PSMNet and also outperforms the iterative RAFT-Stereo and IGEV-Stereo baselines. Compared with IGEV-Stereo, the strongest iterative baseline in EPE, BSEF-Stereo reduces EPE from 0.47 px to 0.41 px and D1 from 2.47% to 2.27%, corresponding to relative improvements of 12.8% and 8.1%. The simultaneous reduction in EPE and D1 is important because it shows improvement in both overall pixel-wise accuracy and difficult outlier regions. These results are consistent with the design objective of combining enhanced feature representation, receptive-field-adaptive updating, and final error-aware correction.

**KITTI 2012 dataset:** As shown in Table 7, BSEF-Stereo performs consistently across all the evaluated thresholds. At the >2 px threshold, its errors are slightly higher than those of DS-Stereo and IGEV++, indicating that small residual deviations remain in some regions. However, BSEF-Stereo achieves the lowest all-region errors at >3 px and >4 px, with 1.32% and 1.02%, respectively, and ties for the best non-occluded >3 px result at 0.98%. For the stricter >5 px metric, it obtains 0.63% in non-occluded regions and 0.84% in all regions, remaining within 0.01 percentage points of the best results. This pattern suggests that the method is especially effective at preventing moderate errors from developing into large disparity failures, including in occluded regions represented by the all-pixel metrics.

**Figure 5:**
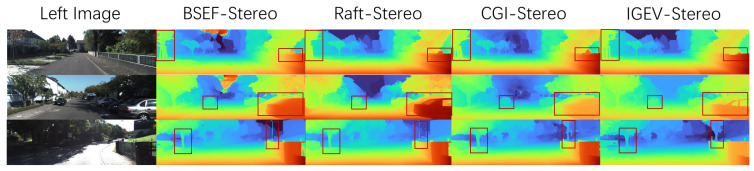
Examples of disparity maps using BSEF-Stereo, RAFT-Stereo, CGI-Stereo, and IGEV-Stereo on the KITTI 2012 test set. Red boxes mark regions selected for close visual comparison, and the color maps encode disparity magnitude.

Figure 5 compares disparity maps produced by BSEF-Stereo, RAFT-Stereo, CGI-Stereo, and IGEV-Stereo on KITTI 2012. In the highlighted regions, BSEF-Stereo produces sharper object boundaries, more complete thin structures, and smoother background surfaces. In particular, discontinuities around vehicles and roadside structures are less diffuse, while large background regions remain spatially coherent. These observations complement the threshold-based results in Table 7 by showing that the reduction in medium and large errors is associated with improved local structure and fewer conspicuous disparity discontinuities.

**KITTI 2015 dataset:** Table 8 shows that BSEF-Stereo achieves the lowest All D1-bg, All D1-all, and Noc D1-bg errors among the compared methods, with values of 1.27%, 1.48%, and 1.19%, respectively. Compared with RegStereo, which ranks second in All D1-all, BSEF-Stereo lowers this metric from 1.50% to 1.48%. Although the numerical margin is small, the improvement is obtained while retaining a lower runtime than RegStereo. Its foreground errors are less competitive: BSEF-Stereo obtains 2.58% All D1-fg and 2.40% Noc D1-fg compared with 2.43% and 2.31% for the best-performing methods in the corresponding columns. It also obtains 1.54% Noc D1-all, whereas RegStereo achieves 1.29%. The contrast between strong background performance and weaker foreground performance suggests that broad contextual aggregation is effective for large relatively stable regions, whereas small moving objects, narrow structures, and partially occluded foreground regions require more targeted modeling.

**Figure 6:**
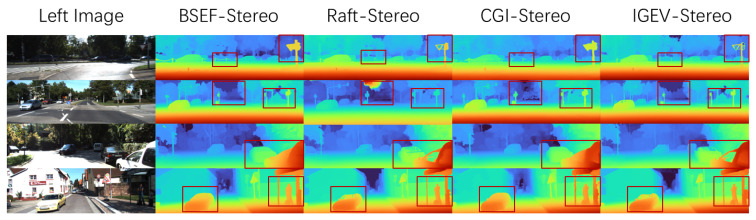
Examples of disparity maps using BSEF-Stereo, RAFT-Stereo, CGI-Stereo, and IGEV-Stereo on the KITTI 2015 test set. Red boxes mark regions selected for close visual comparison, and the color maps encode disparity magnitude.

Figure 6 provides qualitative comparisons with RAFT-Stereo, CGI-Stereo, and IGEV-Stereo. BSEF-Stereo generally produces clearer boundaries and more complete local structures in the highlighted regions, with fewer irregular disparity transitions across background surfaces. At the same time, some small foreground regions remain difficult to reconstruct completely. This visual behavior is consistent with the quantitative gap between the strong D1-bg results and the comparatively weaker D1-fg results, providing a more balanced view of the model’s strengths and remaining limitations.

**Middlebury dataset:** Middlebury contains high-resolution indoor scenes with weakly textured regions, large disparity variations, and fine object structures. As shown in Table 9, BSEF-Stereo achieves the best results among the selected methods, with bad 2.0 errors of 6.7% at half resolution and 5.6% at quarter resolution. Relative to NMRF-Stereo, these values represent absolute reductions of 1.1 and 1.9 percentage points or relative reductions of 14.1% and 25.3%, respectively. The larger improvement at quarter resolution suggests that the iterative updater remains effective when the available spatial detail is reduced, while the strong half-resolution result demonstrates that the method can also exploit finer image structures. Together with the KITTI results, this performance supports generalization across outdoor driving scenes and high-resolution indoor environments.

**Figure 7:**
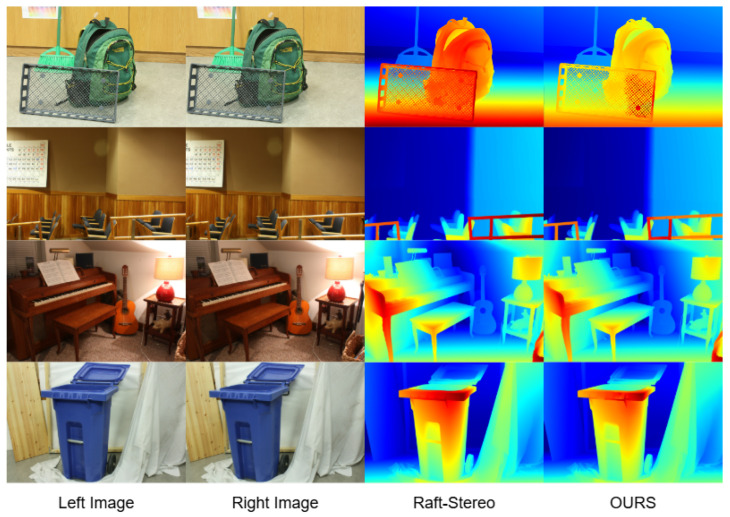
Examples of disparity maps produced by BSEF-Stereo and RAFT-Stereo on the Middlebury dataset. The color maps encode disparity magnitude.

The qualitative comparison in Figure 7 is consistent with the numerical results. Compared with RAFT-Stereo, BSEF-Stereo produces sharper boundaries and smoother local surfaces, particularly around furniture contours and small indoor objects. The improvement is also visible in weakly textured planar regions, where the proposed method generates fewer fragmented or locally inconsistent disparities. These examples suggest that the combination of broader contextual updating and image-guided refinement is useful for balancing surface smoothness with boundary preservation in indoor scenes.

**Figure 8:**
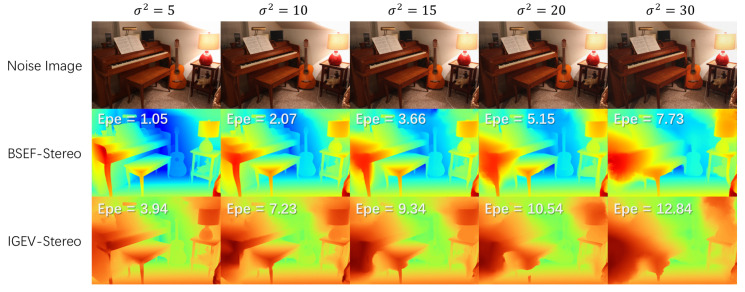
Robustness comparison between BSEF-Stereo and IGEV-Stereo on a Middlebury 2014 image corrupted by Gaussian noise. The color maps encode disparity magnitude; the labels and overlays are legible and do not affect scientific interpretation.

Figure 8 and Table 10 present a preliminary robustness comparison on Middlebury 2014 images corrupted by Gaussian noise.

Both methods degrade as the noise level increases, confirming that corrupted local appearance remains a major challenge for stereo correspondence. Nevertheless, BSEF-Stereo consistently produces lower errors than IGEV-Stereo. At $\sigma^{2}=5$, it reduces bad 2.0 from 36.02% to 11.99% and EPE from 9.99 px to 2.27 px, corresponding to relative reductions of 66.7% and 77.3%. Under the strongest tested noise level, $\sigma^{2}=20$, BSEF-Stereo still reduces bad 2.0 from 68.54% to 40.86% and EPE from 18.11 px to 10.78 px, with relative reductions of approximately 40% for both metrics. The visual examples similarly show that the main disparity structure remains more recognizable for BSEF-Stereo as noise increases. However, the absolute error growth at higher noise levels also shows that the model is not invariant to severe corruption. The analysis therefore remains preliminary because it considers only Gaussian noise; future evaluation should include blur, illumination changes, compression artifacts, and real-world sensor distortions.

## 5. Conclusions

This paper presents BSEF-Stereo, an iterative stereo matching framework that combines channel–position attention, receptive-field-adaptive recurrent updating, and error-aware refinement. Experiments on Scene Flow, KITTI 2012/2015 [13,14], and Middlebury [15] demonstrate competitive accuracy across synthetic, outdoor, and indoor scenes, while the ablation studies verify the complementary contributions of CPA, BGRU, and EAR. In particular, the full model reduces the KITTI EPE from 0.43 px to 0.31 px and achieves strong results on Scene Flow, KITTI 2015 [14], and Middlebury [15], indicating improved disparity estimation in challenging regions.

The current model remains less effective on some small or occluded foreground objects, and the robustness evaluation is limited to Gaussian noise. Future work will therefore focus on lightweight refinement, foreground-aware optimization, and broader evaluations under realistic image degradations.

## Figures and Tables

**Table 1:** Ablation study of the proposed components in BSEF-Stereo.

Model	Attention			Recurrent Update		Refinement		Scene Flow		KITTI	
	CA	PA	CPA	GRU	BGRU	Hourglass	EAR	EPE (px)	D1 (%)	EPE (px)	D1 (%)
Baseline				✓		✓		0.56	2.61	0.43	1.22
+CA	✓			✓		✓		0.50	2.44	0.38	1.12
+PA		✓		✓		✓		0.52	2.52	0.40	1.21
+CPA			✓	✓		✓		0.48	2.39	0.36	1.02
+BGRU					✓	✓		0.47	2.41	0.36	0.98
+EAR				✓			✓	0.50	2.53	0.40	1.18
+CPA + BGRU			✓		✓	✓		0.43	2.36	0.33	0.86
+CPA + EAR			✓	✓			✓	0.45	2.38	0.34	0.92
Full Model			✓		✓		✓	0.41	2.27	0.31	0.72
Note: A checkmark indicates that the corresponding component is used in the model variant.											

**Table 2:** Performance with different numbers of iterations.

Iterations	Scene Flow		KITTI	
	EPE (px)	D1 (%)	EPE (px)	D1 (%)
2	0.953	4.683	0.534	1.784
4	0.613	3.401	0.491	1.162
8	0.495	2.547	0.348	0.813
16	0.454	2.401	0.332	0.787
32	0.413	2.269	0.312	0.724

**Table 3:** Sensitivity analysis of kernel-size settings on Scene Flow.

Kernel Sizes	Scene Flow	
	EPE (px)	>1 px (%)
1×1+1×5	0.42	5.22
3×3+1×5	0.42	5.05
1×1+3×3	0.41	5.08

**Table 4:** Sensitivity analysis of the residual scaling factor ϵ on Scene Flow.

ϵ	Scene Flow	
	EPE (px)	>1 px (%)
0.25	0.42	5.17
0.50	0.41	5.05
0.75	0.47	5.45

**Table 5:** Complexity and efficiency comparison of model variants.

Variant	Baseline GRU	HG	CPA	BGRU	EAR	Params (M)	Peak GPU Memory (GiB)
Baseline (B.)	✓	✓	–	–	–	13.14	1.225
B. w/o GRU/HG + BGRU	–	–	–	✓	–	12.95	1.242
+CPA	–	–	✓	✓	–	12.95	1.242
+EAR (Full)	–	–	✓	✓	✓	15.29	1.251

**Table 6:** Quantitative evaluation of BSEF-Stereo on Scene Flow. Best and second-best accuracy results are highlighted in bold and underlined, respectively.

Method	EPE (px)	D1 (%)
PSMNet [16]	1.09	–
GANet [18]	0.84	4.52
CFNet [30]	0.97	4.70
RAFT-Stereo [11]	0.65	2.92
CGI-Stereo [31]	0.64	2.55
DLNR [12]	0.48	2.36
IGEV-Stereo [20]	0.47	2.47
BSEF-Stereo (Ours)	**0.41**	**2.27**

**Table 7:** Quantitative evaluation of BSEF-Stereo on the KITTI 2012 dataset. Best and second-best accuracy results are highlighted in bold and underlined, respectively. The time column is provided for reference.

Method	>2 px (%)		>3 px (%)		>4 px (%)		>5 px (%)		Time (s)
	Noc	All	Noc	All	Noc	All	Noc	All	
CRE-Stereo [10]	1.72	2.18	1.14	1.83	1.07	1.34	0.83	1.04	0.32
ACVNet [32]	1.83	2.34	1.13	1.47	0.86	1.12	0.71	0.91	0.20
IGEV-Stereo [20]	1.71	2.17	1.12	1.44	0.88	1.12	0.73	0.94	0.18
Any-Stereo [33]	1.75	2.24	1.18	1.52	0.94	1.20	0.78	1.00	0.32
Guard-Net [34]	2.02	2.57	1.22	1.58	0.92	1.19	0.74	0.96	0.03
MoCha-Stereo [35]	1.64	2.07	1.06	1.36	0.80	1.03	0.66	**0.83**	0.03
NMRF-Stereo [36]	1.59	2.07	1.01	1.35	0.78	1.03	0.64	0.84	0.09
IGEV++ [37]	1.56	2.03	1.04	1.36	0.81	1.06	0.67	0.88	0.28
DS-Stereo [38]	**1.49**	**2.02**	**0.98**	1.34	0.76	1.03	**0.62**	0.85	0.35
BSEF-Stereo (Ours)	1.58	2.06	**0.98**	**1.32**	**0.73**	**1.02**	0.63	0.84	0.29

**Table 8:** Quantitative evaluation of BSEF-Stereo on the KITTI 2015 dataset. Best and second-best accuracy results are highlighted in bold and underlined, respectively. The time column is provided for reference.

Method	All (%)			Noc (%)			Time (s)
	D1-bg	D1-fg	D1-all	D1-bg	D1-fg	D1-all	
PCVNet [39]	1.68	3.19	1.93	1.56	3.25	1.80	0.05
CFNet [30]	1.54	3.56	1.88	1.43	2.98	1.73	0.18
ACVNet [32]	1.51	3.80	1.89	1.36	3.09	1.72	0.48
Lac+GANet [6]	1.44	2.83	1.67	1.44	2.83	1.63	1.80
GM-Stereo [40]	1.49	2.96	1.77	1.34	2.97	1.61	0.17
Guard-Net [34]	1.53	3.34	1.83	1.41	3.15	1.70	0.03
Any-Stereo [33]	1.44	3.04	1.70	1.30	2.88	1.56	0.34
IGEV++ [37]	1.31	2.54	1.51	1.20	2.54	1.42	0.28
MoCha-Stereo [35]	1.36	**2.43**	1.53	1.24	2.42	1.44	0.34
RegStereo [41]	1.30	2.54	1.50	1.20	**2.31**	**1.29**	0.37
BSEF-Stereo (Ours)	**1.27**	2.58	**1.48**	**1.19**	2.40	1.54	0.29

**Table 9:** Quantitative comparison on the Middlebury dataset. Best and second-best results are highlighted in bold and underlined, respectively.

Method	Middlebury	
	Half Bad 2.0 (%)	Quarter Bad 2.0 (%)
PSMNet [16]	26.9	20.0
GANet [18]	20.3	11.2
CFNet [30]	15.3	9.8
DLNR [12]	9.5	7.6
Graft-GANet [42]	9.8	–
DCVSMNet [43]	8.1	9.0
NMRF-Stereo [36]	7.8	7.5
BSEF-Stereo (Ours)	**6.7**	**5.6**

**Table 10:** Quantitative robustness comparison on Middlebury 2014 images corrupted by Gaussian noise.

Noise Variance $\sigma^{2}$	BSEF-Stereo		IGEV-Stereo	
	Bad 2.0 (%)	EPE (px)	Bad 2.0 (%)	EPE (px)
5	11.99	2.27	36.02	9.99
10	22.29	4.63	52.01	13.90
20	40.86	10.78	68.54	18.11

## Data Availability

The datasets used in this study are publicly available. The Scene Flow dataset is available at Scene Flow Datasets (https://lmb.informatik.uni-freiburg.de/resources/datasets/SceneFlowDatasets.en.html (accessed on 30 June 2026)). The KITTI 2012 and KITTI 2015 datasets are available at KITTI Vision Benchmark Suite (https://www.cvlibs.net/datasets/kitti/ (accessed on 30 June 2026)). The Middlebury stereo dataset is available at Middlebury Stereo Datasets (https://vision.middlebury.edu/stereo/ (accessed on 30 June 2026)).
